# The attentional-relevance and temporal dynamics of visual-tactile crossmodal interactions differentially influence early stages of somatosensory processing

**DOI:** 10.1002/brb3.210

**Published:** 2014-01-23

**Authors:** Christina Popovich, W Richard Staines

**Affiliations:** Department of Kinesiology, University of Waterloo200 University Ave. W, Waterloo, Ontario, N2L 3G1, Canada

**Keywords:** Attention, crossmodal, ERPs, event-related potentials, sensorimotor integration, somatosensory cortex, tactile, visual

## Abstract

**Background:**

Crossmodal interactions between relevant visual and tactile inputs can enhance attentional modulation at early stages in somatosensory cortices to achieve goal-oriented behaviors. However, the specific contribution of each sensory system during attentional processing remains unclear. We used EEG to investigate the effects of visual priming and attentional relevance in modulating somatosensory cortical responses.

**Methods:**

Healthy adults performed a sensory integration task that required scaled motor responses dependent on the amplitudes of tactile and visual stimuli. Participants completed an attentional paradigm comprised of 5 conditions that presented sequential or concurrent pairs of discrete stimuli with random amplitude variations: 1) tactile-tactile (TT), 2) visual-visual (VV), 3) visual-tactile simultaneous (SIM), 4) tactile-visual delay (TVd), and 5) visual-tactile delay (VTd), each with a 100 ms temporal delay between stimulus onsets. Attention was directed to crossmodal conditions and graded motor responses representing the summation of the 2 stimulus amplitudes were made.

**Results:**

Results of somatosensory ERPs showed that the modality-specific components (P50, P100) were sensitive to i) the temporal dynamics of crossmodal interactions, and ii) the relevance of these sensory signals for behaviour.

**Conclusion:**

Notably, the P50 amplitude was greatest in the VTd condition, suggesting that presentation of relevant visual information for upcoming movement modulates somatosensory processing in modality-specific cortical regions, as early as the primary somatosensory cortex (SI).

## Introduction

It is well-known that attention can modulate neurophysiological responses in modality-specific cortices including: visual (Motter 1993; Gazzaley et al. 2007; Andersen et al. 2008), auditory (Woldorff et al. 1993; Jäncke et al. 1999; Petkov et al. 2004), and somatosensory cortices (Josiassen et al. 1990; Hsiao et al. 1993; Johansen-Berg et al. 2000; Staines et al. 2002). However, recent investigations have begun to examine whether attention influences neural responses across sensory modalities when sensory input from more than one modality is present. Behavioral studies have shown that crossmodal input can also improve performance as indexed by faster reaction times (Hershenson 1962; Gielen et al. 1983), improved detection of weak stimuli (Frens and Van Opstal 1995; Driver and Spence 1998; McDonald et al. 2000), and improved sensory-perception of illusory effects such as the ventriloquist or McGurk illusions (Howard and Templeton 1966; McGurk and MacDonald 1976). Human and animal studies have shown that the mere presence of additional sensory input even when it is irrelevant for performance of a task can enhance neural excitability in the attended sensory modality (Calvert et al. 1997; Macaluso et al. 2000, 2002; Calvert 2001; Foxe et al. 2002; Kayser et al. 2005, 2007; Pekkola et al. 2006; Lehmann et al. 2006; Lakatos et al. 2007; Meehan and Staines 2009), suggesting that interactions between modality-specific cortical representations exist. By contrast, other studies have shown crossmodal enhancement in modality-specific sensory cortex only occurs when both stimuli events are relevant for behavior (Dionne et al. 2010, 2013). These findings suggest that crossmodal processing is likely governed by both bottom-up sensory-sensory interactions and top-down attentional mechanisms in order to allow for the selection, amplification, and integration of sensory input relevant for initiating goal-oriented responses. Bottom-up interactions can occur when salient stimuli from an unattended sensory modality influence neural excitability in the attended modality, while top-down processing occurs when attention is voluntarily directed toward relevant stimuli in the presence of environmental distracters. However, while both these attentional mechanisms can modulate neural responses in modality-specific sensory cortex, it remains unclear how these attentional mechanisms interact during sensory processing of crossmodal stimuli.

Neurophysiological research in the primary auditory cortex of monkeys has provided evidence that sensory-to-sensory interactions exist. Recent studies have shown that neural responses in regionally distinct areas of the primary auditory cortex are enhanced when visual and/or tactile stimuli are paired with auditory stimuli (Kayser et al. 2005, 2007). Lakatos et al. (2007) showed that presentation of somatosensory stimuli increased auditory neural responses when the two stimuli were simultaneously combined versus when the auditory stimulus was presented in isolation. Furthermore, Bizley et al. (2007) reported a 15% neuronal increase in the ferret primary auditory cortex following simultaneous presentation of visuo-auditory stimuli (Bizley et al. 2007). Neuroimaging studies in humans complement the sensory-to-sensory interactions reported in animal findings by showing that the presence of crossmodal input can modulate neural excitability in modality-specific sensory cortices. For example, several functional magnetic resonance imaging (fMRI) studies have reported increased blood oxygenation level-dependent (BOLD) responses in modality-specific cortices due to the mere presence of stimuli from another modality. These interactions have been found between: visual and auditory cortices (Calvert et al. 1997; Calvert 2001; Lehmann et al. 2006; Pekkola et al. 2006), auditory and somatosensory cortices (Foxe et al. 2002; Schürmann et al. 2006), as well as visual and somatosensory cortices (Macaluso et al. 2000, 2002). However, a recent fMRI study investigated crossmodal effects on BOLD responses generated in the primary somatosensory cortex (SI) when both stimuli were relevant for guiding a motor response. Here, relevant unimodal (visual or tactile) and crossmodal stimuli (simultaneous visual + tactile) were presented and participants were required to summate both stimuli by squeezing a pressure-sensitive bulb. In order to ensure that stimulus associations were successfully learned prior to testing, participants completed a brief sensorimotor training session that required them to judge the amplitude of visual and vibrotactile stimuli and make a graded motor response representing the perceived amplitude of the stimuli. Results showed that the greatest BOLD responses were elicited in SI during crossmodal versus unimodal interactions suggesting that combining visual-tactile information relevant for behavior enhances modality-specific excitability in SI (Dionne et al. 2010). In a follow-up study, Dionne et al. (2013); used electroencephalography (EEG) and the same sensory-to-motor task to investigate the time course of crossmodal effects in SI. Results showed that crossmodal interactions between vibrotactile and visual stimuli enhanced the amplitude of the somatosensory P50 component, generated in SI, at contralateral parietal electrode sites only when both stimuli were task-relevant. By contrast, the amplitude of the P100, likely generated in SII, increased bilaterally at parietal electrode sites during presentation of crossmodal stimuli but was not sensitive to the task-relevance of the stimuli. These findings suggest that crossmodal modulation occurs at very early stages in the somatosensory processing stream if both stimuli are relevant for behavior (Dionne et al. 2013).

Several other EEG studies support the finding that crossmodal stimuli can modulate neural excitability at very early stages of sensory processing. For example, Giard and Peronnet (1999); found that visual modulation for audio-visual stimuli, occurred as early as 40-msec post stimulus onset, while audio-tactile modulation has been found at 50 msec (Foxe et al. 2000; Molholm et al. 2002). Kennett et al. (2001); found modulation of visual event-related potentials (ERPs) by irrelevant but spatially aligned tactile stimuli at approximately 140-msec post visual onset, while McDonald et al. (2000); reported modulation of visual ERPs was possible with spatially aligned auditory stimuli. In summary, crossmodal interactions can improve behavioral performance and enhance neural excitability at early stages in modality-specific cortices to achieve goal-oriented behaviors (Dionne et al. 2010, 2013). However, the specific contribution of each sensory system during attentional processing in modality-specific sensory cortices remains unclear. In this study, we manipulated the attentional relevance and temporal onsets of visual and tactile stimuli to examine whether both top-down and bottom-up mechanisms can modulate early stages of somatosensory processing.

The specific aim of this study was to explore the relative contributions of visual priming (bottom-up sensory input) and task-relevance (top-down attention) on influencing early somatosensory cortical responses, namely the P50 somatosensory ERP generated in SI. We hypothesized that somatosensory activity would be modulated based on the temporal onset and stimulus order of task-relevant crossmodal (visual-tactile) events. To test whether bottom-up sensory-sensory interactions influence crossmodal modulation of the P50 component, we manipulated the temporal onsets of visual and tactile events in two crossmodal conditions. In one condition, visual stimuli preceded tactile stimuli by 100 msec to examine whether the presentation of relevant visual information prior to tactile information influenced crossmodal modulation of the P50 component. In the other condition, tactile stimuli preceded visual stimuli by 100 msec. This condition acted as a control to the previously described condition since the onset of the P50 component would have already occurred prior to the presentation of visual information, thus P50 modulation in this case would not be due to crossmodal influences. If bottom up and top-down mechanisms influence early somatosensory ERPs in contralateral SI, then the P50 amplitude should be greatest for relevant crossmodal interactions where visual information preceded tactile information and smallest for the irrelevant unimodal interactions.

## Material and Methods

### Participants

EEG was collected from 20 self-reported right-handed healthy participants (mean age = 26, 10 males). Five subjects were excluded due to either excessive artifacts found during inspection of the raw EEG collection, or the absence of clearly defined somatosensory ERPs of interest (i.e., P50 and/or P100 components). The final sample consisted of 15 healthy participants (mean age = 27.5, 7 men). Experimental procedures were approved by the University of Waterloo Office of Research Ethics. All subjects provided informed written consent.

### Behavioral paradigm

**Figure 1:**
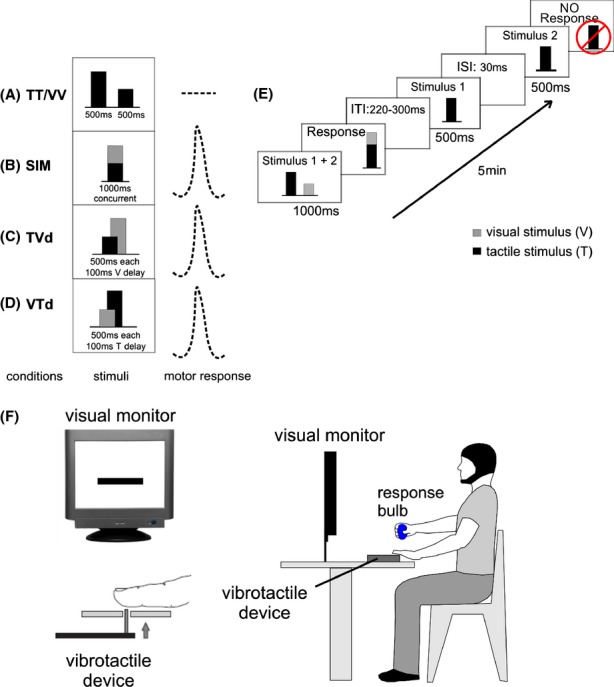
Experimental paradigm. (A) shows the unimodal conditions (VV, TT), (B) shows the crossmodal condition with simultaneously presented visual-tactile stimuli, (C) shows the crossmodal condition where tactile stimuli are presented 100 msec before visual stimuli (TVd), (D) shows the crossmodal condition where visual stimuli are presented 100 msec before tactile stimuli (VTd) between visual-tactile condition (VT). Participants were required to ignore all unimodal conditions and only respond to the crossmodal conditions. To depict the behavioral task, the columns are intended to represent examples of the temporal onset and amplitudes of stimulus events while the dotted trace is a schematic of the corresponding force applied to the squeeze-bulb when making the motor response to those stimuli. (E) shows an example of a bimodal simultaneous condition (SIM) and a unimodal tactile-tactile condition (TT). Subjects were to attend only to bimodal conditions and make a graded motor response with a pressure bulb representing the summation of each stimuli. (ITI; intertrial interval, ISI; interstimulus interval). (F) The experimental setup is depicted for the positioning of participants to receive the tactile and visual stimulation.

The behavioral paradigm consisted of five conditions that presented pairs of discrete visual and/or tactile stimuli with random amplitude variations. Stimuli were always presented in pairs, either sequentially (unimodal conditions) or simultaneously (crossmodal conditions): (1) tactile-tactile (TT; 500 msec each, 30-msec interstimulus interval [ISI]), (2) visual-visual (VV; 500 msec each, 30-msec ISI), (3) visual-tactile simultaneous (SIM; 1000 msec concurrent), (4) visual-tactile with a 100-msec temporal delay between stimulus onsets (visual-tactile delay, [VTd]; 500 msec each, visual presented first), and (5) tactile-visual with a 100-msec temporal delay between stimuli (tactile-visual delay, [TVd], tactile presented first) (refer to Fig. 1A–D). Participants were instructed to only attend to the crossmodal stimuli (i.e., TT/VV conditions were ignored), judge the amplitude of the two stimuli, and then make a graded motor response representing the sum of these amplitudes by squeezing a pressure-sensitive bulb with their right hand (Fig. 1E). Prior to the EEG collection, participants underwent a 5-min training session with visual feedback in a sound attenuated booth to learn the relationship between the amplitudes of the stimuli and the corresponding force required to apply to the bulb. During training, a horizontal target bar appeared on the computer monitor and subjects were instructed to squeeze the pressure-sensitive bulb with enough force to raise another visual horizontal bar to the same level as the target bar. At the same time, as subjects applied force to the bulb with their right hand the vibrotactile device vibrated against the volar surface of their left index finger with corresponding changes in amplitude. In other words, as they squeezed harder on the bulb the amplitude of the vibration increased proportionately. Subjects were instructed to pay attention to these changes in amplitude as they related to the force they were applying to the bulb. This training allowed subjects to become familiar with the relationship between the vibrotactile stimulus amplitude and the corresponding force applied to the bulb. To control for force related trial to trial differences, stimulus amplitudes were scaled such that no single stimulus required a squeeze of more than 25% of an individual's maximum force, thus the response for adding two stimuli was never more than 50% of an individual's maximum force. Stimuli were always presented in pairs, either unimodally (two visual or two tactile) presented sequentially, or crossmodally (one visual and one tactile), presented simultaneously or with a 100-msec temporal offset between each stimuli.

### Experimental paradigm

During the experiment, participants sat comfortably in a sound attenuated booth and were instructed to visually fixate on the computer monitor, rest the volar surface of their left index finger gently on the vibrotactile device, and hold the pressure-sensitive response bulb in their right hand (Fig. 1F). Participants were instructed to attend only to crossmodal interactions, judge the amplitude of both the visually presented horizontal bars and the vibrotactile stimuli, and produce force graded motor responses using the pressure-sensitive bulb that represented the summation of both stimulus amplitudes. Stimuli were presented for 1 sec after which participants were required to make their motor response immediately following presentation of the crossmodal stimuli during a 2.5 sec window prior to the start of the next trial, for a total of 3.5 sec per trial. Each condition was randomized and performed in six blocks of 120 trials with each block lasting approximately 5 min. The order of the conditions was counterbalanced across each block and all subjects performed the same six blocks in sequential order.

### Stimuli

Visual stimuli consisted of a centrally presented horizontal bar (6 cm wide), which raised to varying heights on a computer monitor positioned 50 cm in front of the subject and represented different visual amplitudes. Vibrotactile stimuli consisted of discrete vibrations delivered by a custom made vibrotactile device applied to the volar surface of the left index finger. Vibrotactile stimulation was controlled by converting digitally generated waveforms to an analog signal (DAQCard 6024E; National Instruments, Austin, TX) and then amplifying the signal (Bryston 2BLP, Peterborough, Ontario, Canada) using a custom program written in LabVIEW (version 8.5; National Instruments). Varying the amplitude of the driving voltage to the vibrotactile device produced proportional changes in vibration of the device on the finger. The amplitude of each discrete vibration was constant within a trial and varied randomly between trials. The average stimulus amplitude across all trials including a tactile stimulus did not differ between the experimental conditions. The frequency of the vibration was held constant at 25 Hz. Participants received 70 db whitenoise (Stim2; Neuroscan, Compumedics USA, Charlotte, NC) throughout the training session and the experiment to prevent auditory perception of the vibrotactile stimulus.

### Data acquisition and recording parameters

EEG data were recorded from 64 electrode sites (64-channel Quick-Cap, Neuroscan, Compumedics USA) in accordance with the international 10–20 system for electrode placement, and referenced to the linked mastoids (impedance <5 kOhms). EEG data were amplified (20,000×), filtered (DC-200 Hz), and digitized at 500 Hz (Neuroscan 4.3, Compumedics USA) before being saved for subsequent analysis. Individual traces were visually inspected for artifacts (i.e., blinks, eye movements, or muscle contractions) and any contaminated epochs were eliminated before averaging. On average a minimum of at least 80 trials per condition were analyzed for each participant.

**Figure 2:**
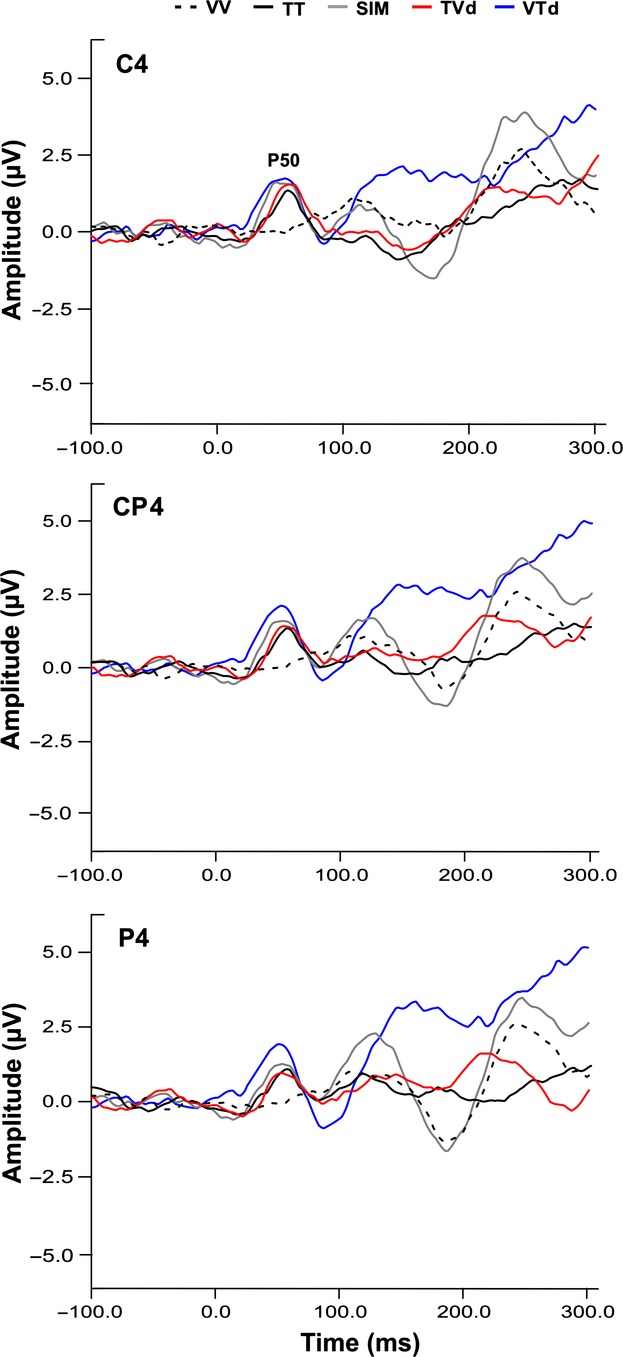
Grand averaged P50 waveforms. Grand average waveforms all for conditions are shown for parietal electrode sites contralateral to vibrotactile stimulation (C4, CP4, P4). The P50 ERP component is labeled on the trace for electrode site C4. Blue, red, and gray traces show VTd, TVd, SIM conditions while black solid and dashed traces show TT and VV conditions, respectively.

**Figure 3:**
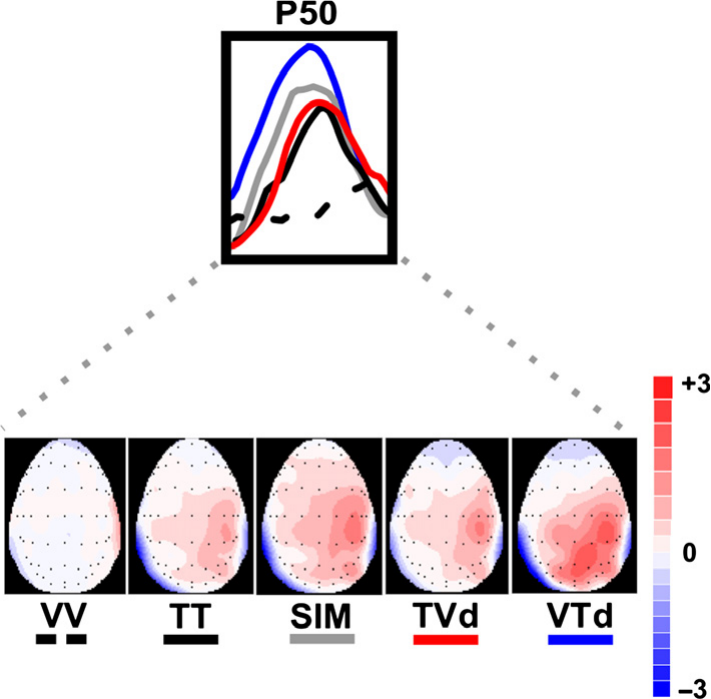
Scalp topography maps of the P50 component. Inset shows modulation of the P50 ERP waveforms in response to bimodal and unimodal conditions. The P50 ERP component is labeled on the trace for electrode site CP4. Blue, red, and gray traces show VTd, TVD, SIM conditions while black solid and dashed traces show TT and VV conditions, respectively. Below images show group averaged data of peak areas of cortical activity generated over a 30-msec time window (40–70 msec) centered around the P50 ERP peak. All values are in microvolts (μV).

**Figure 4:**
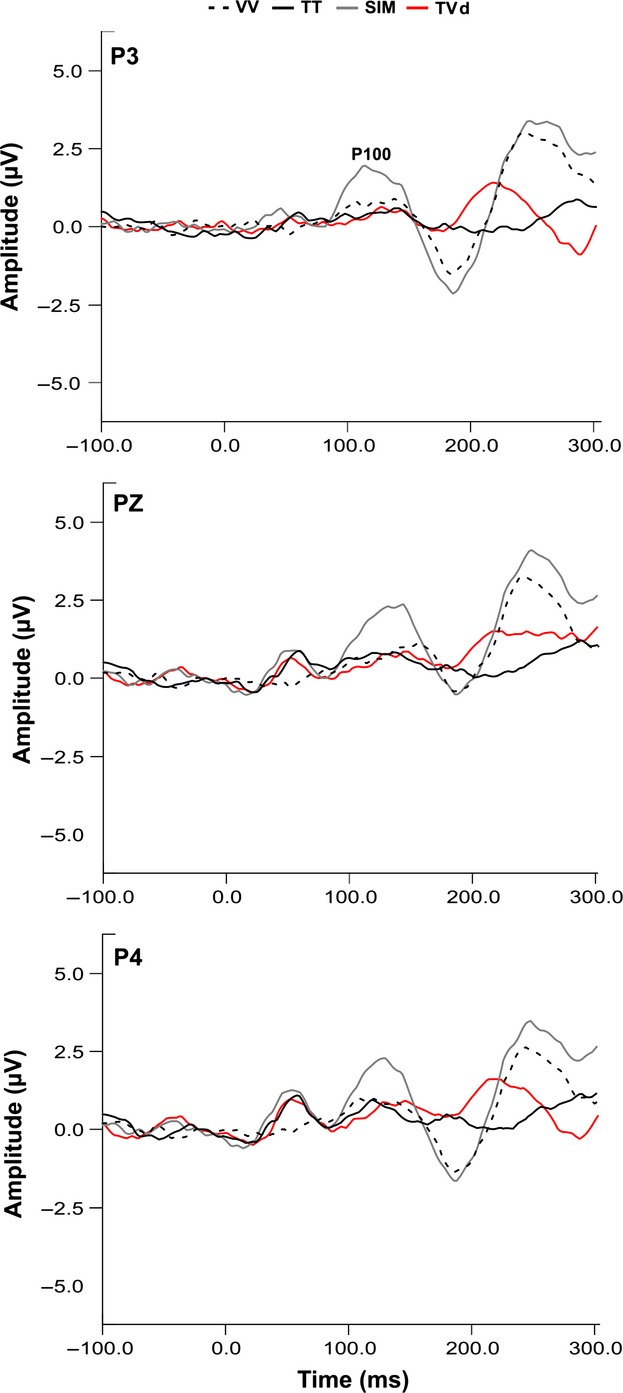
Grand averaged P100 waveforms. Grand average P100 waveforms are shown for parietal electrode sites (P3, PZ, P4) for SIM, TVd, and TT conditions. The P100 ERP component is labeled on the trace for electrode site P3. Gray, red, and black traces show SIM, TVd, TT, and VV conditions, respectively.

Event-related potentials were averaged to the onset of each stimulus relative to a 100-msec pre-stimulus baseline. Somatosensory ERPs were measured from individual participant averages for each task condition. Mean ERP amplitudes and latencies were computed for each subject within specified time windows selected around the post stimulus latencies of early somatosensory ERP components: P50 (40–70 msec), P100 (90–125 msec). Figures 2 and 4 illustrate the distribution of these potentials over parietal electrode sites. Figure 3 illustrates the voltage distribution across the scalp at the latency of the P50. On the basis of these topographies, the amplitude of each potential was measured from pre-selected electrode sites corresponding to scalp locations showing maximal voltage during the corresponding latency window. Thus, the P50 component was measured from sites centered at CP4 (C4, CP4, P4), roughly overlying right sensory-motor cortex and contralateral to the vibrotactile stimulus. The P100 is typically observed bilaterally at parietal electrode sites thus amplitude and latency of this component was measured from P3, PZ, and P4. All amplitudes were measured as raw voltage relative to the pre-stimulus baseline.

### Data analysis

#### ERP data analysis

To test the hypothesis that the temporal onset and stimulus order of task-relevant crossmodal (visual-tactile) events would contribute to the modulation of early modality-specific somatosensory ERPs, a one-way repeated measures analysis of variance (ANOVA) with condition as a factor was carried out on the amplitude and latency of the P50 component at electrode sites C4, CP4, and P4 (regions contralateral to vibrotactile stimulation). These ANOVAs were followed by a priori contrasts performed to test the hypothesis that modulation of the P50 would be greatest for the task-relevant crossmodal visual-tactile task with a 100-msec temporal delay between stimulus onsets (VTd) and smallest for the irrelevant unimodal tactile-tactile (TT) task. Our statistical approach to the P100 component had to exclude analysis of the VTd condition since the 100-msec temporal delay between the visual and tactile stimuli produced an interaction with the visual ERPs over the time window (90–125 msec) chosen for the P100 peak amplitude. A one-way repeated measures ANOVA with condition as a factor was also computed on the amplitude and latency of the P100 at electrodes sites P4, PZ, and P3. Tukey's post hoc tests were carried out on any main effects to investigate whether relevant crossmodal conditions would be associated with greater amplitudes compared to the irrelevant unimodal conditions.

#### Behavioral data analysis

Behavioral data were analyzed by summing the amplitudes of the two target stimuli and comparing this to the amplitude of the response that is the force applied to the pressure-sensitive bulb. The percent difference between the summed target stimulus amplitude and the actual response amplitude was calculated and a one-way repeated measures ANOVA was conducted to assess statistical differences across the experimental conditions.

## Results

### P50 ERPs

**Figure 5:**
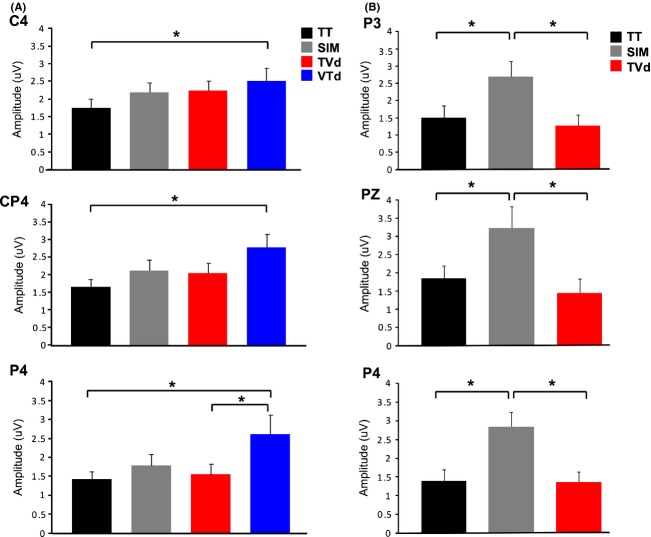
Group ERP means. Group means for (A) P50 and (B) P100 ERP components. Blue bars represent group data for the crossmodal condition where presentation of visual stimuli preceded tactile stimuli (VTd), red bars represent group data for the crossmodal condition where presentation of tactile stimuli preceded visual stimuli (TVd), gray bars represent group data for the crossmodal condition where visual + tactile stimuli were presented simultaneously, black bars represent group data for the unimodal tactile condition (TT). Error bars show SEM, *Denotes significance *P* < 0.05. (A) Mean P50 amplitude measured at C4, CP4, P4, (B) depicts the mean P100 amplitude at P3, PZ, P4, respectively.

All subjects demonstrated a clear P50 component (mean latency 53 ± SE 2 msec) in response to vibrotactile stimuli presented to the left index finger. Figure 2 shows the grand averaged waveforms for all conditions at electrode sites C4, CP4, and P4 approximately overlying contralateral somatosensory cortex (centered at CP4). Scalp topography maps representing group averaged data were created by averaging neural responses generated over the 30 msec time window (40–70 msec) centered around the P50 peak to observe task-specific differences in cortical modulation (refer to Fig. 3). As illustrated in Figure 2, all conditions including vibrotactile stimuli (i.e., TT, SIM, TVd, VTd) elicited robust neural activity in somatosensory regions contralateral to stimulation. Notably, the VTd condition also elicited robust activation in modality-specific visual cortex, while the VV condition showed minimal activation overall. Statistical results using a one-way repeated measures ANOVA showed a main effect of condition on the modulation of the P50 amplitude at electrode CP4 (*F*_3,42_ = 2.81, *P* = 0.05) as well as a trend toward significance for electrode P4 (*F*_3,42_ = 2.49, *P* = 0.07), but no effect at electrode C4 (*F*_3,42_ = 1.53, *P* = 0.22). A priori contrasts showed that modulation of the P50 amplitude was greater in the VTd condition compared to the TT condition for all three electrode sites (C4(*F*_1,14_ = 4.44, *P* = 0.041; CP4 (*F*_1,14_ = 8.20, *P* = 0.007); P4(*F*_1,14_ = 6.20, *P* = 0.017)). It was also shown that P50 amplitude was significantly greater in the VTd versus the TVd condition at electrode P4 (*F*_1,14_ = 4.87, *P* = 0.033) with a strong trend toward significance for the same effect at CP4 (*F*_1,14_ = 3.37, *P* = 0.07) (refer to Fig. 5A). Analysis of the P50 latency using a one-way repeated measures ANOVA revealed a main effect of conditions at electrodes CP4 (*F*_3,42_ = 3.08, *P* = 0.04) and P4 (*F*_3,42_ = 3.52, *P* = 0.02). Tukey's post hoc analysis on these electrodes both showed that the latency of the P50 amplitude occurred earlier in the VTd condition than the TT condition (VTd mean latency = 50 msec versus TT mean latency = 57 msec). No main effect of condition was found at electrode C4 (*F*_3,42_ = 2.19, *P* = 0.1).

### P100 ERPs

The P100 component was present in all conditions with vibrotactile stimulation. However, we omitted analysis of the VTd condition since the fixed temporal delay of 100 msec between the visual and tactile stimuli created an interaction whereby the visual ERPs overlapped the specified time window of 90–125 msec centered around the P100 peak amplitude. As seen in Figure 4, the grand averaged P100 waveforms (mean latency 118 ± 4 msec) for the remaining three conditions (SIM, TVd, TT) displayed a bilateral distribution at parietal sites and maximal amplitude at electrode site PZ. Results showed a main effect of condition observed at electrode sites P4 (*F*_2,28_ = 7.95, *P* = 0.002), PZ (*F*_2,28_ = 5.97, *P* = 0.007), and P3 (*F*_2,28_ = 10.73, *P* < 0.001). Tukey's post hoc tests showed that for each electrode site, the amplitude of the P100 was larger in the SIM compared to the TVd task (*P* < 0.05) and the TT task (*P* < 0.05, Fig. 5B). A main effect of condition was found for the P100 latency at electrode P4 using separate one-way repeated measures ANOVA (*F*_2,28_ = 3.64, *P* = 0.04). However, Tukey's post hoc analysis revealed no statistically significant differences between conditions. Furthermore, no main effect of condition was found for electrodes PZ (*F*_2,28_ = 1.02, *P* = 0.37) or P3 (*F*_2,28_ = 0.36, *P* = 0.7).

### Behavioral data

**Figure 6:**
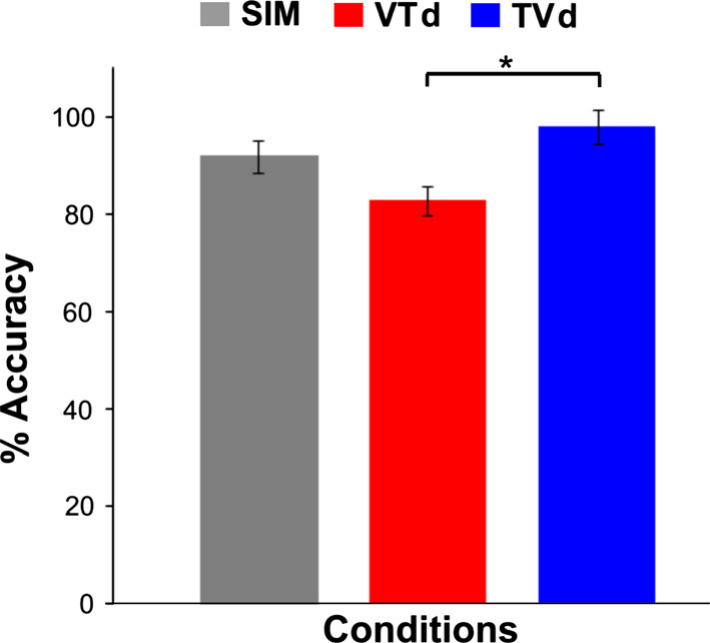
Group means for behavioral data. The gray bar graph represents group data for the visual + tactile simultaneous condition (SIM), the red bar graph represents group data for the condition where tactile stimuli were presented 100 msec before visual stimuli (TVd), and the blue bar graph represents group data for the condition where visual stimuli are presented 100 msec before tactile stimuli (VTd) between visual-tactile condition (VT). Error bars show SEM.

Figure 6 shows the behavioral means and standard error bars for each task-relevant crossmodal condition: SIM (mean = 92, SE = 3.3), VTd (mean = 83, SE = 2.9), TVd (mean = 98, SE = 3.4). A one-way repeated measures ANOVA was performed on the error differences represented as a percent score across all conditions and showed that there was a main effect of condition (*F*_2,16_ = 8.45, *P* = 0.003). Post hoc Tukey's test showed that performance in the VTd condition was significantly different than the TVd task. Participants tended to produce lesser force than the ideal target in the VTd condition. There were no other differences between conditions.

## Discussion

In this study, we used EEG and crossmodal stimuli (visual + vibrotactile) to examine the roles of visual information and attentional relevance in modulating early cortical responses generated in SI. To test the influence of bottom-up sensory-sensory interactions and top-down attentional processes on early modality-specific cortical responses, we devised a novel experimental protocol that manipulated the temporal onsets of task-relevant crossmodal (visual + tactile) interactions. In one condition, visual stimuli preceded the onset of tactile stimuli by 100 msec (i.e., VTd), in order to observe the influence of the visual modality on the P50 component generated in SI. In another condition, tactile stimuli preceded the onset of visual stimuli by 100 msec (i.e., TVd), in which case, the P50 would have been elicited prior to the onset of visual information and modulation would not reflect crossmodal effects. We hypothesized that both bottom-up interactions and top-down attentional mechanisms influence early somatosensory ERPs, whereby, modulation (mainly of the P50 component) would be greatest for the relevant crossmodal condition where visual events occurred 100 msec prior to tactile events (VTd), and smallest, for irrelevant tactile unimodal condition (TT). Our results confirmed our hypotheses by showing that early somatosensory ERPs, namely the P50 and P100 components were sensitive to (i) the temporal dynamics of crossmodal interactions, and (ii) the relevance of these sensory signals for behavior. Specifically, modulation of the P50 amplitude depended on the temporal onset of crossmodal stimuli with the greatest effects seen when visual events preceded tactile events (VTd condition), followed by similar modulation between the other crossmodal conditions (SIM and TVd), and lastly the smallest modulation was seen for the irrelevant unimodal tactile condition (TT). As expected, there was no P50 modulation for the unimodal visual condition (VV) since no tactile events occurred and no behavioral response was required.

It is of particular importance to highlight the differences in P50 modulation between the crossmodal conditions. In crossmodal conditions with a 100 msec temporal delay between the onset of visual and tactile stimuli (VTd and TVd conditions), we showed that P50 modulation was greater in the VTd condition relative to the TVd condition. This finding was expected since in the TVd condition, the P50 component would have already occurred before presentation of the visual information. Our topographic maps (Fig. 3) complement our P50 results by showing that only conditions including vibrotactile stimulation (i.e., TT, SIM, TVd, VTd) elicited neural activation in somatosensory regions contralateral to stimulation, while the VV condition showed minimal activation overall. However, a prominent difference in neural activity specific to the VTd condition was revealed, whereby robust neural activation was elicited not only in somatosensory cortex but in visual areas as well. These results imply that presentation of relevant visual information for upcoming movement modulates somatosensory processing as early as SI. Moreover, the lack of SI activity seen in the VV condition implies that the activation of the visual cortex during the VTd condition was not simply due to volume conduction via additional sensory input, but instead, was specific to the task-relevance of the visual information in performing goal-oriented behavior. Lastly, the amplitude of the P100 component was enhanced during the SIM condition and suppressed during the TVd condition and TT condition. This finding suggests that enhancement of the P100 component depended on the attentional relevance and temporal alignment of visual-tactile events. Overall, this study shows that early somatosensory ERPs generated in modality-specific cortical regions are modulated by both bottom-up sensory interactions between visual and somatosensory modalities and top-down attentional influences. Thus, both the attentional requirement and the neural networks that control modality-specific sensory processing are necessary for crossmodal interactions to occur (Dionne et al. 2013).

The P50 component is a somatosensory ERP observed maximally in parietal cortices near the post-central sulcus contralateral to tactile stimulation, and typically varies in latency between 40 and 60 msec post stimulus onset (Desmedt et al. 1983). It can be elicited via somatosensory stimuli (tactile, vibratory, peripheral nerve stimulation) in most subjects whereby changes in the amplitude of the response are believed to reflect changes in SI excitability (Allison et al. 1989; Zhu et al. 2007). However, the precise role of the P50 component in processing somatosensory information remains elusive. It has been suggested that the P50 component reflects a preattentional inhibitory filter mechanism critical for sensory gating of irrelevant stimuli, and the integrity of higher order functions (Freedman et al. 1987, 1991; Jerger et al. 1992; White and Yee 2006). Studies in patient populations support this theory with findings showing diminished P50 gating in neurological illnesses associated with inhibitory control deficits including: Alzheimer's dementia (Thomas et al. 2010), posttraumatic stress disorder (Karl et al. 2006), schizophrenia (Adler et al. 1982; Patterson et al. 2008), and bipolar I disorder (Schulze et al. 2007; Lijffijt et al. 2009). However, Schubert et al. (2008) suggested that the modulation of the P50 is dependent on the attentional demands of a task, such that tasks with higher degrees of difficulty are more successful in driving facilitation of the P50 amplitude. If this supposition is true, then enhancement of P50 component may instead reflect cognitive strategies applied during perceptual stages of sensory processing whereby relevant sensory signals are amplified via thalamo-cortical gating mechanisms (Yingling and Skinner 1976; Desmedt and Tomberg 1989; Brunia 1993), before they can be relayed to higher order association cortices for further processing.

The P100 component has a relatively broad scalp distribution and is thought to be generated in bilateral secondary somatosensory cortex (SII) (Hari et al. 1984, 1983; Mima et al. 1998; Zhu et al. 2007). Bilateral activation is typically maximal over contralateral posterior parietal electrode sites and somewhat less robust at ipsilateral sites (Desmedt and Robertson 1977; Desmedt and Tomberg 1989; Hämäläinen et al. 1990). The P100 is similar to the P50 component, in that it is elicited by tactile and vibratory stimuli (Goff et al. 1977), and is modulated by attention (Desmedt et al. 1983; Michie 1984; Michie et al. 1987; Josiassen et al. 1990; Eimer and Forster 2003; Kida et al. 2004; Schubert et al. 2006). Selective attention studies have reported increased P100 amplitudes in attended versus unattended tactile stimuli with effects being greater than earlier ERP responses generated in SI (Josiassen et al. 1982; Desmedt et al. 1983; Josiassen et al. 1990; Bolton and Staines 2011). Overall, attention influences both the P50 and P100 amplitudes, but modulatory changes may be related to differences in experimental paradigms used and/or psychological factors (Desmedt and Robertson 1977; Goff et al. 1977).

### Attentional modulation in somatosensory cortex

Studies investigating the effects of sustained tactile-spatial attention have shown that attention to task-relevant versus irrelevant spatial locations enhances processing of tactile stimuli and modulates somatosensory cortex (SI and SII) (Desmedt and Robertson 1977; Michie 1984; Michie et al. 1987). Several functional neuroimaging studies have found that sustained spatial attention to one hand versus the other during bilateral tactile stimulation enhances hemodynamic responses within contralateral SI and sensorimotor regions (Macaluso et al. 2000; Meador et al. 2002). A positron emission tomography study reported that the anticipation of tactile stimulation can increase activity in contralateral SI even in the absence of any stimuli (Roland 1981). Furthermore, EEG investigations comparing somatosensory ERPs elicited by tactile stimulation applied to the hands, have reported that attending to the location of tactile stimulation modulates both early and late somatosensory ERPs (N80, P100, N140) with increased amplitudes for the attended versus unattended tactile location (Desmedt and Robertson 1977; Michie 1984; Michie et al. 1987; Garcia-Larrea et al. 1995). However, SI responses as early as 45–50 msec post stimulus onset have been reported using an attentional vigilance task (Zopf et al. 2004). Notably, a recent study using simultaneous EEG and fMRI recordings found that sustained spatial attention during bilateral tactile stimulation (Braille) modulated early somatosensory ERPs (P50, N80, P100, and the long latency potential (LLP)) as well as increased BOLD signals in SI, SII, the inferior parietal lobe and frontal areas. Correlation results showed that attentional modulation of SI was found to be positively correlated with attentional effects for the P50 and the LLP components (Schubert et al. 2006). The LLP component has multiple neural generators from broadly distributed locations, and is often seen as a sustained positivity occurring approximately 200–500 msec post stimulus (Hämäläinen et al. 1990; Michie et al. 1987). The precise role of this later positivity remains unclear; however, several attention-based tactile ERP studies have implied that the LLP may share functional similarities to the P300 component, such that increases in the LLP amplitude is thought to reflect the amount of attentional resources devoted to a given task (Desmedt and Robertson 1977; Desmedt and Tomberg 1989; Michie et al. 1987). These findings imply that sustained tactile attention modulates neural activity generated in SI at both early and later stages of tactile processing (Schubert et al. 2008).

### Crossmodal input modulates somatosensory cortex

It is well-documented that attention modulates modality-specific sensory cortex, however, little is known about how multiple sensory inputs across modalities are integrated for purposeful goal-oriented behaviors. Recently, researchers have begun to investigate how attention operates across sensory modalities with examination focused on the crossmodal links between touch and vision. Eimer and Driver (2000) used a tactile-spatial attention task whereby participants were required to attend and respond to target stimuli presented to the primary modality (touch) while ignoring distractor stimuli presented at the unattended hand and stimuli shown in the task-irrelevant modality (vision). Results showed enhanced somatosensory ERPs to tactile stimuli presented at the attended locations and increased modulation of early visual ERPs elicited by irrelevant visual stimuli presented at task-relevant tactile locations. These findings suggest that sustained attention to one modality can influence neural excitability in another spatially congruent modality (Eimer and Driver 2000). In a behavioral study, it was reported that visualization of the finger improved acuity judgments of tactile gratings applied to the fingertip (Taylor-Clarke et al. 2004), while a separate EEG study showed modulation of somatosensory ERPs as early as 80 msec post-stimulus when participants viewed stimulation of their own arm (Taylor-Clarke et al. 2002). In another EEG study, Meehan and Staines (2009) examined crossmodal effects on somatosensory evoked potentials elicited via median nerve stimuli. Results showed that enhancement of P50 amplitude was greatest when crossmodal stimuli (visual + vibrotactile) were presented in spatiotemporal alignment but attention was directed only to vibrotactile events. These results suggest that the presence of visual information that is spatiotemporally congruent to relevant tactile information enhanced the amplitude of the P50 component. However, it was uncertain if participants were aware that crossmodal events were synchronous, therefore, alterations in cognitive strategy to perform the task are unknown (Meehan and Staines 2009). Lastly, Dionne et al. (2013) showed that the amplitude of P50 was sensitive to simultaneous presentation of crossmodal stimuli, but only when both crossmodal events were relevant for behavior, and not when one event was irrelevant (i.e., when participants only responded to one modality). Specifically, the presence of visual stimuli, alone, did not enhance the P50 amplitude, suggesting that modulation of this component is mediated by top-down sensory gating mechanisms.

Results also showed that enhancement of the P100 amplitude was greatest during simultaneous presentation of crossmodal (visual + vibrotactile) stimuli relevant for behavior versus task-irrelevant unimodal stimuli. Despite these P100 results and the findings reported in this study, crossmodal effects on this component are variable, and seem to depend on the spatial location of attention. For example, studies using EEG and sensory oddball tasks have investigated crossmodal links in spatial attention between vision and touch. In tactile manipulations, participants responded to tactile ‘oddball’ targets at attended spatial locations (primary modality) while ignoring visual stimuli (secondary modality). Results showed that attended, relative to unattended tactile stimuli, enhanced the negativity of the somatosensory N140 component, but failed to produce attentional effects at earlier stages of somatosensory processing (Eimer and Driver 2000). However, recent work by Jones and Forster (2013) showed that engaging in a visual task while performing an exogenous tactile attention task diminished cortical modulation at early stages of somatosensory processing. Here, subjects either performed a tactile exogenous attention task while either just watching a visual stream of letters (single task), or were required to perform the tactile task and detect targets within the visual stream (dual task). ERP results showed diminished modulation of the N80 and P100 somatosensory components during the dual task suggesting that early stages of somatosensory processing are sensitive to crossmodality effects (Jones and Forster 2013). Plausible explanations for the inconsistent crossmodal effects on early stages of somatosensory processing may be differences in the attentional tasks employed (i.e., crossmodal sensory integration task versus tactile spatial attention task), and/or in the attentional demands required between studies (i.e., graded force response representing the summation of visual and tactile stimuli with the hand versus vocal response made when target stimuli were presented at attended spatial locations) (Eimer and Driver 2000; Eimer 2001; Dionne et al. 2013; Jones and Forster 2013).

Crossmodal interactions between relevant sensory inputs can facilitate perceptual processing in modality-specific sensory cortex to achieve goal-oriented behaviors. Studies have shown that the presence of an additional (but task-irrelevant) modality can enhance neural excitability in the attended modality (Calvert et al. 1997; Macaluso et al. 2000, 2002; Calvert 2001; Foxe et al. 2002; Kayser et al. 2005, 2007; Pekkola et al. 2006; Schürmann et al. 2006; Lehmann et al. 2006; Lakatos et al. 2007; Meehan and Staines 2009), suggesting that attention within one modality can modulate neural excitability (to some extent) in another sensory modality. Furthermore, recent neuroimaging studies have found that relevant crossmodal stimulation (i.e., tactile and visual sensory input) increases both neurophysiological responses in SI relative to unimodal stimulation (i.e., either visual or tactile sensory input) (Dionne et al. 2010, 2013). Taken together, these studies suggest that both bottom-up (i.e., the presence of an additional sensory modality) and top-down attentional mechanisms (i.e., task-relevance) work together to process and integrate relevant sensory signals for successful execution of goal-oriented behaviors. However, the neural mechanisms underpinning the contribution of each sensory system during crossmodal attentional processing remains unclear. In this study, we examined the relative contribution of visual information in modulating early somatosensory ERPs by manipulating the temporal parameters of relevant visual-tactile interactions. Results showed that modulation of the P50 component varied based on the temporal delay between relevant bimodal stimuli, with greatest enhancement seen when visual information occurred 100 msec prior to the onset of tactile information. In addition, the P100 component was enhanced during simultaneous bimodal interactions relevant for behavior, but not during bimodal interactions where tactile information occurred 100 msec prior to visual information, or during irrelevant unimodal interactions suggesting that the P100 component was increased only when visual-tactile events occur in temporal synchrony and require selective attention. Lastly, behavioral results revealed differences between the sensory-motor responses produced during the VTd versus the TVd conditions, such that, participants tended to over-squeeze the pressure-sensitive bulb when summating TVd stimuli. It is plausible that participants may have employed different cognitive strategies to facilitate processing of these crossmodal conditions. It certainly is possible that such modulation of these modality-specific regions would have some behavioral benefits in terms of the efficient sensorimotor transformation. However, since participants were not explicitly asked whether they used a specific strategy to aid their sensorimotor judgments, we can only speculate potential factors that may have produced the differences in behavior found in our study.

There are some notable limitations in the design of the experimental paradigm used in this study which must be considered. Although the crossmodal conditions with 100-msec temporal delays between the onset of visual or tactile stimuli events (i.e., TVd and VTd), were advantageous for interpreting crossmodal effects on the P50 component, the temporal delay interfered with the timing of some early (i.e., the P100 component for the VTd condition) and all later onset ERPs (i.e., N140) beyond typical latency boundaries, thus crossmodal effects could not be discussed for these components. Second, the behavioral results of this study suggest that participants may develop different cognitive strategies in order to facilitate perceptual processing of crossmodal stimuli with temporal delays between the onsets of each stimulus. Previous studies using the same stimuli described in this study have reported no differences in behavior during unimodal (TT, VV) conditions versus simultaneous presentation of crossmodal (visual + vibrotactile) conditions, suggesting that performance accuracy was similar across all conditions (Dionne et al. 2010, 2013). Indeed, the discrepancy between these behavioral results compared to the results of this study reveal a need for future studies to investigate if a potential relationship between these early changes in neural excitability and behavioral responses exists.

Notwithstanding these limitations, the results of this study are novel and suggest that presentation of visual information relevant for upcoming sensory-guided movement can facilitate tactile processing at very early stages in SI. Our findings complement previous observations reporting that crossmodal attention effects can occur at early stages in modality-specific sensory ERP components (Eimer and Driver 2000; Taylor-Clarke et al. 2002; Dionne et al. 2013). Notably, this study extends the current literature by showing that crossmodal modulation of early somatosensory ERPs is facilitated by bottom-up sensory interactions between visual-tactile cortical associations and top-down sensory gating mechanisms. Overall, this research offers novel and important information about how the brain merges sensory input from multiple modalities in order to execute goal-oriented behaviors.
